# *QuickStats*: Percentage* of Adults Who Ever Used an E-cigarette^†^ and Percentage Who Currently Use E-cigarettes,^§^ by Age Group — National Health Interview Survey, United States, 2016^¶^

**DOI:** 10.15585/mmwr.mm6633a6

**Published:** 2017-08-25

**Figure:**
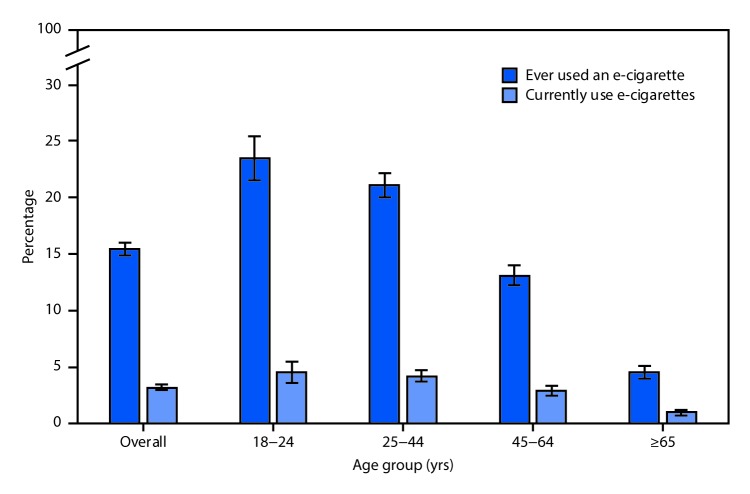
Overall, 15.4% of adults aged ≥18 years had ever used an e-cigarette, and 3.2% currently used e-cigarettes in 2016. Adults aged 18–24 years were the most likely to have ever used an e-cigarette (23.5%); the percentage declined steadily to 4.5% among adults aged ≥65 years. Adults aged 18–24 years (4.5%) and 25–44 years (4.2%) were more likely to be current e-cigarette users than adults aged 45–64 years (2.9%) and those aged ≥65 years (1.0%). Across all age groups, fewer than one fourth of adults who had ever used an e-cigarette reported being a current user.

**Source:** National Health Interview Survey, 2016 data. https://www.cdc.gov/nchs/nhis.htm.

