# *Erratum:* Vol. 66, No. 33

**DOI:** 10.15585/mmwr.mm6644a11

**Published:** 2017-11-10

In “*QuickStats*: Percentage of Adults Who Ever Used an E-cigarette and Percentage Who Currently Use E-cigarettes, by Age Group — National Health Interview Survey, United States, 2016,” on page 892, the caption should have read as follows:

“Overall, **15.3%** of adults aged ≥18 years had ever used an e-cigarette, and 3.2% currently used e-cigarettes in 2016. Adults aged 18–24 years were the most likely to have ever used an e-cigarette (**23.8%**); the percentage declined steadily to **4.4%** among adults aged ≥65 years. Adults aged 18–24 years (**4.7%**) and 25–44 years (4.2%) were more likely to be current e-cigarette users than adults aged 45–64 years (**2.8%**) and those aged ≥65 years (1.0%). Across all age groups, fewer than one fourth of adults who had ever used an e-cigarette reported being a current user.”

